# Healing beyond the physical: Ethical approaches to emotional support in post-stroke recovery

**DOI:** 10.4102/ajod.v15i0.1909

**Published:** 2026-05-15

**Authors:** Olukoya A. Olotu, Anthea Rhoda

**Affiliations:** 1 Department of Physiotherapy, Faculty of Health Sciences, University of the Free State, Bloemfontein, South Africa

**Keywords:** biomedical ethics, counselling psychology, emotional support, post-stroke recovery, rehabilitation

## Abstract

Stroke is one of the major contributors to long-term disability in the world, with significant emotional and psychological implications for survivors. While physical rehabilitation is often prioritised in post-stroke recovery, the emotional impact, such as grief, anxiety, depression, and identity disruption, remains under-addressed, particularly in a developing country such as South Africa. This article argues for a more ethical, grounded and holistic approach to stroke recovery that prioritises emotional support alongside physical treatment. Drawing on the disciplines of counselling psychology and rehabilitation science, the article explores how interdisciplinary approaches can enhance the emotional needs of stroke patients. Through a conceptual methodology, this article critiques the limitations of biomedical ethics when applied to stroke rehabilitation and proposes a complementary ethical model, such as care and narrative ethics. These frameworks emphasise empathy, relationship-building and meaning-making as central components of ethical care. Through theoretical analysis, the article demonstrates how patients’ emotional narratives can inform ethical patient-centred rehabilitation interventions. The article also deliberates the implications for healthcare providers, offers practical recommendations and outlines policy integration strategies for embedding emotional care within rehabilitation programmes. By highlighting the ethical significance of emotional support, the study offers a more compassionate, culturally sensitive and inclusive model of stroke care in line with medical humanities and the future of healthcare delivery.

## Introduction

Stroke is a major global public health issue that is among the leading causes of chronic disability around the world (Avan & Hachinski 2021; Khandelwal & Gupta 2023). Millions are affected by stroke every year, with long-term physical, cognitive and emotional rehabilitation being the consequences for many survivors (Feigin et al. 2022). Despite considerable advances in neurorehabilitation and technological improvements in physical function and motor skills, the psychosocial and emotional aspects of stroke recovery have received less attention in clinical practice (Hartley, Burger & Inglis-Jassiem 2022). This is concerning as the impact of stroke on mental health has been associated with reports of depression, anxiety, emotional lability, social withdrawal and identity disruption (Towfighi et al. 2017). The emotional aspect of health is not secondary but an integral part of wholeness and overall well-being, and therefore, has a significant impact on full rehabilitation outcomes (Eticha et al. 2025).

Globally, health care systems are centred on biomedical frameworks that prioritise clinical effectiveness, disease control and measurable outcomes (Hayman et al. 2023). Consequently, emotional and psychological stress that comes with stroke recovery is often less emphasised. While the approach may prove effective in clinical settings, it fails to address the complex, subjective experiences in post-stroke recovery (Smythe et al. 2022). In rehabilitation settings, especially in under-resourced health care systems like South Africa, patients often experience inadequate services, overwhelmed healthcare personnel with limited access to psychosocial support (Maart et al. 2024). In these contexts, emotional care is often neglected and not considered an ethical necessity.

The World Health Organization and United Nations Children’s Fund (2022) highlight the role of integrated, person-centred care as a hallmark of high-quality health systems. Thus, stroke rehabilitation should not be centred solely on bodily recovery but on personhood wholeness, which includes emotional resilience, psychological meaning-making and social reintegration (Kingau et al. 2024). This approach entails an ethical shift from predominantly biomedical interventions to one that encompasses relationality, empathy and narrativity. Medical humanities, informed by literature, philosophy, ethics and psychology, among others, provide a foundation for such an ethical reorientation (Roest, Milota & Leget 2021).

In South Africa, systemic inequalities, historical trauma, cultural diversity and inadequately resourced healthcare facilities persist across the country (Ntsiea 2019). Most rehabilitation centres are overwhelmed, and counselling services are either inadequate or not accessible to many across the population (Booysen & Kagee 2022). In addition, cultural diversity may cause or complicate the delivery of emotional care. For example, cultural beliefs of many traditional Africans, that is, people with indigenous African beliefs as opposed to Western culture, stress relationships within the community, spirituality and mind-body connections and experiences that may not align indigenously with the Western biomedicalised lens (Letsoalo et al. 2024). These contextual realities call for a culturally appropriate approach, multidisciplinary model, localised traditional practices and values that incorporate global ethical standards.

This article aims to contribute to the ongoing debate on ethical stroke care by advocating for a holistic, emotionally responsive model of rehabilitation. This conceptual article is based on the interdisciplinary literature from counselling psychology, rehabilitation sciences and medical humanities. Through this lens, the article seeks to understand how emotional support can be ethically integrated into stroke care and to critique current biomedical approaches that fail to address emotional needs (McCaul et al. 2020).

## Background: Biomedical ethics and its limits

Biomedical ethics has been the regulatory foundational framework guiding clinical decision-making in global healthcare systems (Forte, Kawai & Cohen 2018; Torrorey-Sawe 2025). The four main principles of biomedical ethics being applied in medical education, policy and clinical practice are autonomy, beneficence, non-maleficence and justice. While these principles have played a major role in safeguarding patient rights and developing professional accountability, they have also been questioned for their inadequacies, especially in long-term emotional care settings like stroke rehabilitation (Beauchamp & Childress 2019). Critics argue that biomedical ethics incline towards individualistic and rationalistic approaches at the expense of social and cultural contexts and, in most cases, fail to account for the diversity and complexity of patients (Russo 2021). This model does not sufficiently capture how relationships, community dynamics and socio-cultural interactions inform patients’ ethical decisions. Hence, relational ethics is more applicable than individual autonomy; considering stroke survivors often rely on their caregivers or family members in their recovery (Gómez-Vírseda, De Maeseneer & Gastmans 2019).

Recent studies have called for integrating alternative ethical paradigms that promote care ethics, narrative ethics and virtue ethics, which emphasise empathy, relationality and contextual sensitivity (Juujärvi, Kallunki & Luostari 2020). These ethical frameworks are pertinent to the medical humanities, where the moral aspects of care are considered within a broad interdisciplinary approach. Medical humanities can fill the gaps in biomedical ethics by advocating empathy, support, patient narratives and cultural sensitivity (Mbonde et al. 2024). Though biomedical ethics is a critical part of health care governance, it is inadequate to cater to the emotional and psychosocial needs of stroke survivors. The incorporation of medical humanities into the ethical conversation holds prospects, especially in South Africa, where the challenges are historically and culturally mediated.

## Emotional distress in stroke survivors

The emotional impact of stroke is broad and largely undermined in clinical and ethical frameworks (Broomfield et al. 2024). Although physical consequences of stroke, such as hemiplegia, aphasia or sensory loss, gain much attention in clinical practice, the emotional aspect of stroke is an overlooked aspect in rehabilitation (Shahid, Kashif & Shahid 2023). Most often, stroke survivors experience emotional distress such as depression, anxiety, anger, frustration, sadness and existential despair. The affective and emotional domains are part of the recovery experience and should be given ethical consideration (Ferro & Santos 2020).

In spite of the clear evidence for emotional distress in stroke survivors, there is little standardisation across healthcare systems with regard to emotional assessment (Quinn, Elliot & Langhorne 2018). Rehabilitation programmes usually are limited in terms of time, goal-directed, and often heavily physical outcome-driven (Meng et al. 2020). Additionally, there is a lack of health professionals specifically trained to assess emotional distress for stroke survivors; this, in turn, creates gaps that need to be addressed (McCurley et al. 2019). In sum, emotional distress in stroke survivors is complex and of ethical concern. It involves neurobiology, personal history, cultural meaning and structural inequities. Neglecting this aspect of stroke recovery may lead to fragmented and inadequate care. Hence, emotional assessment and support should be incorporated into the rehabilitation process. This serves not only to improve outcomes but also to reinstate human dignity and restore a faster pathway to recovery.

As outlined in the South African Clinical guidelines, a multidisciplinary approach to post-stroke recovery is essential for full rehabilitation (Sekome 2018). However, emotional assessment, which involves evaluating a person’s state of mind, is not explicitly stated in the guidelines. This gap underscores the need to integrate emotional support into clinical procedures for post-stroke recovery (McCaul et al. 2020).

## Medical humanities lens

Medical humanities is the practice of medicine from a humanistic perspective and involves an interdisciplinary approach to incorporating the humanities into medical education and practice, including literature, philosophy, history, ethics and arts. It seeks to promote effective healthcare by developing compassion, critical thinking, narrative skills and ethical sensitivity among healthcare professionals (eds. Crawford, Brown & Charise 2020). In the stroke recovery context, medical humanities is a useful framework in terms of reconsidering emotional care, critiquing biomedical reductionism and endorsing more compassionate, person-centred health services (McQueen, Mobilio & Moulton 2022).

Narrative is one of the fundamental contributions of medical humanities. Rita Charon’s (2008:39) thoughts of narrative medicine are based on the premise that clinical action ought to entail attentive listening to patients’ stories – they are full of meaning, emotion and identity. For people living with stroke, telling stories can be a way to work through traumatic experiences, re-orient oneself and articulate needs that might not surface in a clinical assessment (Fioretti et al. 2016). Narrative allows clinicians not only to focus on symptoms but also to consider the person beyond the disease, thereby promoting more profound engagement in ethics work (Salifu 2025).

Also, visual, media and performance art can offer ways to comprehend the emotional aspects of stroke patients. Theatre-based interventions can facilitate role playing and emotional expression, while poetry or journaling can support emotional reflection (Chan et al. 2023; Pangestu, Septianingrum & Faizah 2023). These imaginative forms work within narrative and care ethics by giving voice to the patient (Brown et al. 2021).

Alternatively, the visual and performing arts are additional avenues for understanding and expressing the emotional dimensions of illness. However, stroke-related disabilities such as aphasia or decreased motor control may compromise the feasibility of expression through writing or performance (Arora et al. 2026). In these instances, modalities such as music, culturally relevant song traditions, and augmentative and alternative communication (AAC) tools can provide accessible pathways to emotional expression. In many African traditions, communal singing, rhythm and oral storytelling provide cultural mediums through which survivors can reconnect with their identity and community (Kuyler et al. 2025).

In summary, medical humanities emphasise emotional therapy, storytelling and morality as integral aspects of healing. In the process, it enhances the lives of both patients and providers and paves the way for a more compassionate, context-sensitive medicine (Anderson et al. 2024). For societies such as South Africa, where cultural heterogeneity and structural inequalities influence health experiences, medical humanities offer a morally robust and pragmatic strategy toward post-stroke rehabilitation.

## Theoretical framework for ethical emotional care

The ethical integration of emotional care into post-stroke rehabilitation requires a theoretical underpinning. Though biomedical ethics provides the basics, the emotional distress of stroke victims, identity reconstruction and relational interdependence are often unattended (Li et al. 2024:2–3). Three complementary ethical approaches are explored in this section: Care ethics, narrative ethics, and person-centred rehabilitation ethics. Each provides conceptual justification for emotional care as not merely supplementary but central to ethical practice:

**Care ethics:** In care ethics, rehabilitation is not limited to physical recovery but also includes attentiveness, empathy and responsiveness to others’ needs. Applied to stroke rehabilitation, it emphasises that survivors are more than just patients with physical disabilities; they are persons with relationship webs, and these relationships are vital to their recovery process (Scheffler & Mash 2020). Their emotions count as they engage in rehabilitation activities. In practice, the care-oriented approach entails clinicians attending to signs of emotional distress, like withdrawal, mourning and frustration that might otherwise go unnoticed, and deploying interventions that affirm the survivor’s humanity (Melillo et al. 2025). The care ethics strongly echoes cultural philosophies of Ubuntu within the South African context, which emphasise communal obligation and interconnectedness (Dokman 2024).

**Narrative ethics:** Furthermore, narrative ethics propounded by scholars such as Rita Charon (2017) argue that illness is not just a biological barrier, but also a narrative interjection, an intrusion that alters life stories and identity. Narrative ethics promotes storytelling in which stroke survivors share their journeys and experiences in their own unique way. Indeed, some survivors of stroke are frequently silenced by speech impairment and social disconnection. The ability to regain such speech and share their narrative heals beyond physical (Arora et al. 2026).

**Person-centred rehabilitation ethics:** A third compatible framework is person-centred rehabilitation ethics, rooted in humanistic psychology as propounded by Carl Rogers (1995). This approach encourages respect for patients’ values, preferences and goals, where patients engage in a partnership rather than being passive (Santana et al. 2018). In the context of rehabilitation, it implies that ethical practice must be grounded in shared decision-making, in which therapeutic goals are negotiated rather than imposed. According to person-centred therapeutic procedure, recovery goes beyond restoration of motor skills; rather, it entails helping and supporting victims to pursue life goals (Davidson et al. 2017). These theories provide a strong basis for repositioning post-stroke rehabilitation by emphasising relational responsibility, narrative engagement and recognition of individuality. Emotional healing is not secondary to physical recovery but an integral aspect that facilitates holistic being.

In sum, while care ethics, narrative ethics and person-centred rehabilitation ethics have developed from distinct intellectual traditions, they all emphasise relationships, lived experience and the significance of individual context in healthcare (Pangestu et al. 2023). Care ethics highlights moral responsibility in caregiving relationships, narrative ethics emphasises how patients’ stories inform understanding about illness and guide ethical reflection, while person-centred rehabilitation ethics focuses on patients’ values and goals as the basis for planning care. These frameworks strengthen each other by emphasising lived experiences and identity reconstruction in post-stroke recovery (Li et al. 2024).

### Conceptual framework: Ethical emotional support in stroke rehabilitation

**FIGURE 1:**
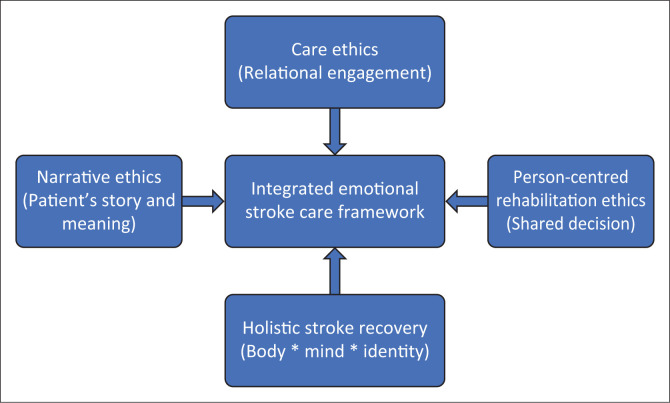
Integrated ethical framework for emotional support in stroke rehabilitation.

Figure 1 illustrates how three ethical perspectives connect to inform emotionally responsive stroke rehabilitation. The model illustrates how care ethics, narrative ethics and person-centred rehabilitation ethics interact in guiding an emotionally responsive stroke rehabilitation. This integration supports relational engagement, narrative recognition, and participatory rehabilitation, contributing to holistic stroke recovery.

## Gaps in current practice

There is increasing awareness about the need for psychological support for stroke survivors; however, globally, the call for emotional support is still limited, especially in low-resource healthcare settings like South Africa (Booysen & Kagee 2022). Rehabilitation professionals, particularly physiotherapists, often have regular contact with stroke survivors during therapy sessions. This positions them to observe or detect emotional changes, such as withdrawal, frustration or a lack of motivation. These facts justify the need for stronger integration of emotional care competence into rehabilitation training and practice (Alexanders & Douglas 2016).

Firstly, one of the most common barriers stems from time constraints and being overloaded with patients. When practicing in busy healthcare systems, whether as a physiotherapist (physical therapist), occupational therapist or speech therapist, the aim is to demonstrate improvement in physical functioning, especially amid limited resources (Gleadhill et al. 2022).

Secondly, a major challenge is the limited training of rehabilitation personnel in emotional care. Most health sciences curricula in South Africa and beyond focus more on the biomedical model, leaving graduates with minimal preparation for emotional and psychosocial aspects of rehabilitation (Hooblaul, Olagbegi & Nadasan 2024). Counselling skills, cultural competency and trauma-informed approaches are rarely included within the training of physiotherapists, occupational therapists or nurses. When professionals feel personally inclined to show empathy towards patients, they may lack the prerequisite skills for responding constructively to expressions of sadness, hopelessness or frustration (Hong 2024). Some rehabilitation professions, including physiotherapy, occupational therapy, and speech therapy, include introductory psychology modules within their early training. The modules seem to be elementary and not enough to cater to the complex emotional needs of stroke survivors. Thirdly, the delivery of stroke rehabilitation services is often weakened as a result of fragmented care pathways. Emotional support, when available, tends to occur concurrently with or separate from physical rehabilitation rather than being integrated into a coordinated framework of care (O’Dell 2023). Hence, the need for ethical approaches for emotional support in post-stroke recovery. Finally, measuring the success of interventions emphasising physical outcomes only diminishes the importance of emotional well-being. Health systems, especially those within resource-stretched environments, depend on objective measures of success such as distance walked, handgrip strength or speech clarity (Njohjam, Falonne & Ngoule 2025). Emotional health, on the other hand, is not easily quantifiable and, as such, is frequently left out of institutional reporting. Patients with a physical ‘successful’ outcome may still live with depression, anxiety or social isolation; these psychosocial experiences of recovery are not visible within the biomedical performance paradigms (Baxter, Burton & Fancourt 2022; Desmet et al. 2025). In South Africa, these challenges are worsened by systemic disparities. Public hospitals and rehabilitation facilities receive proportionally larger, heterogeneous populations with high socio-economic variation for whom provision of equipment remains challenging in the context of staff ratio to patients (Ntsiea 2019).

## Interdisciplinary approaches to emotional support

No singular provider or profession can assume the burden of addressing the emotional aspects of stroke recovery. Rather, it requires an interdisciplinary and transdisciplinary effort that is time-extended and multi-layered, with support from a variety of health professionals (Kingau et al. 2024). This approach shares responsibility across a continuum of care to ensure that survival wholeness receives full attention (Hartley et al. 2022). An interdisciplinary team could consist of psychologists, social workers, physiotherapists, occupational therapists, speech therapists, community health workers, nurses and doctors, all bringing their unique knowledge. While psychologists bring training in emotional assessment and therapeutic intervention, social workers address family dynamics, economic stressors and social reintegration (Bendowska & Baum 2023). Rehabilitation professionals, for instance, physiotherapists and speech therapists, primarily focus on the physical aspects of rehabilitation; however, actively incorporating reflective listening in their daily practice is an important part of emotional support (Makaula, Msomi & Ross 2025). Community health workers thus extend this spectrum of care beyond institutions and into the home and community settings to provide culturally competent support (Van Iseghem et al. 2023). As stated in previous sections, by incorporating narrative, mindfulness, and community partnerships within the interdisciplinary approach, this model is proposed to enable a layered form of care that tackles not only the survivor’s body but also their mind, identity and social world.

## Recommendations for ethical practice

Clinical guidelines need to be updated to reflect emotional assessments and interventions alongside physical evaluations. Such guidelines could require screening for post-stroke depression, routine referral to counselling and inclusion of emotional outcomes in care audits. In practice, emotional screening can be integrated into routine assessments in rehabilitation settings using validated instruments, such as the Stroke Impact Scale (SIS), Patient Health Questionnaire (PHQ-9) or other structured screening processes (Dajpratham et al. 2020). These instruments require little extra time and can be administered during the normal therapy session; thus, clinicians can assess emotional distress while continuing other physical rehabilitative measures. By embedding these tools in standard rehabilitation workflows, emotional care is integrated without adding burden on already resource-constrained settings.

In addition, several suggestions can be implemented to ensure the direct reform and transformation of rehabilitation systems in South Africa and beyond:

**Education and training:** Education and training are the cornerstones of ethical practice. Rehabilitation programmes need to broaden to include modules on emotional care, narrative competence and medical humanities. Training emerging professionals in reflective listening, trauma-informed care and cultural competence can equip graduates to see survivors as complete persons rather than victims of stroke (Shrivastava et al. 2024). Continuous professional development should build on these skills and provide workshops in counselling techniques or interdisciplinary collaboration.

**Policy integration:** Clinical guidelines need to be updated so that emotional assessments and interventions are equally mandated as physical assessments. Such recommendations might require screening for post-stroke depression, referral to counselling by default, and inclusion of emotional outcomes on audit of care (Zhao et al. 2018). By incorporating these mandates into national or institutional policy, emotional care is elevated from optional to standard ethical practice.

**Embracing cultural sensitivity:** Emotional care shouldn’t be transplanted from Western models, but rather be tailored to fit local beliefs, values and traditions. In South Africa, for example, this could include the incorporation of oral storytelling in therapy, acceptance of traditional healers, counselling in local dialect, and involving patients in shared decision-making processes (Letsoalo et al. 2024).

In sum, integrating traditional healers and culturally competent communication practices into rehabilitation pathways might further bolster emotional support. In many South African communities, traditional healers play a trusted role as providers of spiritual guidance, counselling and social mediation. Collaborative engagement, such as referral networks, culturally informed dialogues between biomedical practitioners and traditional healers or community-based rehabilitation initiatives, may improve trust and access to care (Nyembezi, Ngcobo & Lehmann 2024). While the availability of trained interpreters or counselling services in the patients’ preferred languages can enhance the positive impact on emotional assessment and therapeutic communication.

## Implications for South Africa and beyond

The South African environment presents unique challenges and opportunities for integrating emotional care within stroke rehabilitation. Underpinned by limited resources, cultural diversity, and the historical trauma of apartheid, this terrain is on which conventional biomedical models fall short but where medical humanities-based alternatives might flourish (Ntsiea 2019).

South Africa is a country of multiple languages, ethnicities and cultures, each with its own beliefs regarding health, illness and healing. Emotional distress may be communicated culturally through idioms and somatic expression (Moonsamy & Gurayah 2024). Practitioners must be sensitised to these expressions in order to offer ethical care and avoid giving pathologising interpretations through a Western psychological lens. The emotional impact on survivors can be further exacerbated by socio-economic vulnerability, joblessness or systemic oppression in the country. Given the weight of emotional distress, moral duty must be considered beyond clinical application, rather as an issue of social justice (Williams, Osman & Hyon 2023). Supporting survivors’ psychological needs in this context means supporting calls for equitable access to rehabilitation, addressing systemic barriers and enhancing practices that work for every citizen. Applying to the South African context, emotional support should be responsive, flexible, available and culturally relevant. While a lack of resources may be a challenge, it also presents an opportunity to develop new models of care that are community and culture-focused (Letsoalo et al. 2024). By embedding emotional care to wider ethical and social justice endeavours, South Africa has an opportunity to lead the way in harnessing more holistic recovery paradigms that not only result in better clinical outcomes but also move towards healing deeper wounds of marginalisation, disparity and cultural disconnection.

Although this discussion is contextualised in the South African setting, the ethical and practical considerations raised in this article are also relevant to global stroke rehabilitation (Avan & Hachinski 2021). Similar challenges are evident in many health systems, especially in low- and middle-income countries, where limited resources and fragmented rehabilitation pathways are common (Kleinitz et al. 2024). Therefore, the incorporation of relational ethics, narrative approaches and person-centred rehabilitation principles may advance more holistic frameworks of care outside of South Africa. These frameworks thus provide flexible strategies for enhancing restorative rehabilitation practices across diverse healthcare contexts globally, with a focus on emotional support, culturally responsive practices and interdisciplinary collaboration.

## Conclusion

This article argues that emotional support is not peripheral to rehabilitation but central to the ethical care of stroke survivors. The inclusion of medical humanities, such as integration of care ethics, narrative ethics and person-centred rehabilitation ethics, provides the ideas and tools to support the incorporation of emotional care within stroke rehabilitation. These frameworks, care ethics, narrative ethics and person-centred rehabilitation ethics stretch beyond the constraints of biomedical models. They remind us that moral care and story-centred are relational and aimed towards the values and goals of survivors themselves (Kass & Faden 2018). As highlighted in the previous sections, the gaps identified in current biomedical procedures include limited time, inadequate training and fragmented pathways, and call for the inclusion of a new framework that promotes an ethical approach to emotional support in post-stroke recovery (Aderinto et al. 2023). Interdisciplinary and transdisciplinary approaches can offer an applied pathway to delivering emotional care throughout rehabilitation. By engaging psychologists, social workers, community health facilitators and rehabilitation therapists within an interconnected care model, survivors can access layers of support to address the complexity of recovery (Falko et al. 2025).

Narrative interviews, reflective listening, and mindfulness are strategies that can be implemented now and are ethically defensible, especially when carried out through interprofessional teamwork focused on communication and shared decision-making.

The South African context underlines the urgency and desirability of such a vision. Resource limitations and diversity in culture constitute the biggest hurdles but also provide room for creativity. South Africa has the opportunity to lead by integrating medical humanities and culturally situated approaches towards caring that are clinically effective and socially just as well as culturally appropriate. This makes emotional care not only a therapeutic tool but also a moral obligation toward inclusivity, dignity and justice in health services (Matahela 2025). In summary, stroke rehabilitation needs to be conceptualised as an experience that heals more than just physically. Emotional support is endemic to conducting ethical care, and its inclusion demands new perspectives, practices and policy change.
